# Helicobacter pylori Cytotoxin-Associated Gene A (cagA) and Vacuolating Cytotoxin Gene A (vacA) Genotypes in Gastrointestinal Patients From Central Thailand

**DOI:** 10.7759/cureus.64164

**Published:** 2024-07-09

**Authors:** Suchitraporn Sukthaworn, Hathaiwan Moungthard, Chayanit Sirisai, Worapong Anuponganan, Chumpol Peerathippayamongkol, Maneerut Mus-u-dee, Suparat Taengchaiyaphum

**Affiliations:** 1 Division of Research and Technology Assessment, National Cancer Institute, Bangkok, THA; 2 Department of Surgery, National Cancer Institute, Bangkok, THA; 3 Department of Endoscopy, National Cancer Institute, Bangkok, THA; 4 Department of Anatomical Pathology, National Cancer Institute, Bangkok, THA; 5 National Center for Genetic Engineering and Biotechnology, National Science and Technology Development Agency (NSTDA), Bangkok, THA

**Keywords:** vaca gene, helicobacter pylori, genotypes, gastrointestinal diseases, caga gene

## Abstract

Introduction

The development of diseases associated with *Helicobacter pylori* (*H. pylori*) infection is closely linked to its virulence genes, which vary by geographic region. This study aimed to determine the prevalence of *H. pylori* cytotoxin-associated gene A (*cagA*) and vacuolating cytotoxin gene A (*vacA*) genes and their genotypes in patients with gastrointestinal diseases.

Methods

Patients diagnosed with gastrointestinal disease based on endoscopic findings were recruited for the study. Gastric biopsies were collected to screen for* H. pylori* infection using polymerase chain reaction (PCR). Subsequently, infected samples were tested for *cagA *and *vacA* genes, and their genotypes were analyzed by sequencing.

Results

Among 250 cases, 56% (140/250) exhibited gastrointestinal diseases. Of these cases, 32.1% (45/140) were infected with *H. pylori*. Regarding gene detection, 40 (88.9%) samples were positive for *cagA*, while all samples were positive for *vacA*. For *cagA*, the Western type with the ABC pattern was the most prominent. There was a statistically significant association between *cagA* genotypes and clinical outcomes, with the Western type being more prevalent in gastritis patients. For *vacA*, there was a high prevalence of the s1 and i1, while the m1 and m2 showed similar prevalence. In our combined analysis, the dominant *vacA *genotype combinations were s1m1i1 (46.7%). There were no statistical differences between the *vacA *genotypes and clinical outcomes (P > 0.05).

Conclusion

This study revealed a high prevalence of *H. pylori* *cagA* and *vacA* genes, but there were variations in their genotypes. A correlation was observed between the Western-type *cagA* and gastritis; however, no association was found between *vacA* genotypes and clinical outcomes.

## Introduction

*Helicobacter pylori* (*H. pylori*) is a pathogenic bacterium linked to gastrointestinal diseases such as gastritis, peptic ulcer disease (PUD), and gastric cancer (GC). It possesses virulence factors that aid in adhesion, translocation, inflammation, and infection in host epithelial cells. This bacterium exhibits genetic heterogeneity, with polymorphisms in its virulence factors influencing its pathogenicity [1]. Key virulence factors, including cytotoxin-associated gene A (*cagA*) and vacuolating cytotoxin gene A (*vacA*), are crucial determinants of virulence, playing significant roles in disease severity and contributing to the initiation and progression of gastrointestinal diseases, particularly GC [2,3].

*CagA*, encoded by the *cag* pathogenicity island (*cag* PAI), produced a cytotoxic protein. The *cagA* PAI synthesizes a type IV secretion system (T4SS) that injects the oncoprotein cagA into the host epithelial cells, triggering intracellular signaling cascades that lead to inflammatory cytokine production, alterations in cell polarity, and cell proliferation. These responses contribute to the pathophysiology of gastric epithelial cells [4]. Based on a conserved five-amino acid repeat motif (EPIYA motif) in the C-terminal region, CagA protein is classified into Western and East Asian types. EPIYA-A and EPIYA-B are found in both types, while EPIYA-C is specific to the Western type and EPIYA-D to the East Asian type [5]. Determining the type and pattern of the EPIYA motif is more critical than merely detecting *cagA* [6].

The *vacA* gene is the second most effective toxin contributing to the pathogenicity of *H. pylori*. This gene encodes the vacuolating cytotoxin protein, which induces vacuole formation and damages the epithelial cells of the stomach in eukaryotes. *VacA* polymorphisms are predominantly present in three regions: the signal (s1 and s2), middle (m1 and m2), and intermediate (i1 and i2) regions. *Helicobacter pylori* strains carrying the *vacA* s1, m1, and i1 alleles pose a high risk of GC due to increased toxin production and greater vacuolating activity compared with strains carrying the *vacA* s2, m2, and i2 alleles, which are rarely linked to PUD and GC [7]. Different combinations of the *vacA* s, m, and i alleles produce VacA toxins with distinct cytotoxic activity. In vitro experiments demonstrated that strains with the s1m1 genotype exhibit high cytotoxic activity, whereas strains with s2m2 exhibit no cytotoxic activity. The s1m2 may or may not induce cytotoxic activity depending on the presence of the i1 allele [8]. Studies indicate that the *vacA* s1m1 strain is highly correlated with PUD and GC. Additional studies have also found that strains containing the *vacA* i1 allele can increase the risk of GC [9,10].

*Helicobacter pylori* infection is closely associated with the development of gastrointestinal diseases. The *cagA* and *vacA* genes of *H.* *pylori* contribute to more severe and diverse pathogenicity. We aimed to investigate the prevalence and genotypes of these virulence genes in *H. pylori*-infected patients at the National Cancer Institute, located in Central Thailand.

## Materials and methods

Study population and specimens

In this study, we enrolled 250 gastric patients who underwent gastroscopy at the Endoscopy Center of the National Cancer Institute, Thailand, from March to October 2023. Inclusion criteria were as follows: age over 18 years, no prior history of chronic severe medical illness, and provision of informed consent. Key exclusion criteria included the following: recent use of antibiotic treatment or proton pump inhibitors within one month before recruitment, a history of previous *H. pylori *eradication, contraindications for gastric biopsy, and incomplete data. All participants were diagnosed based on endoscopic findings, and those diagnosed with gastrointestinal diseases underwent gastric biopsies from the antrum of the stomach.

DNA extraction

Bacterial DNA was extracted from gastric biopsy tissues using QIAamp® DNA Mini Kit (QIAGEN, Hilden, Germany) following the manufacturer's instructions. The quantity and purity of the total DNA were assessed using a NanoDrop spectrophotometer (Thermo Scientific, Waltham, MA).

Molecular detection of *H. pylori* and its virulence genes

Fifty nanograms of DNA were utilized to detect the presence of *H. pylori* using 16S ribosomal RNA (16S rRNA)-specific primers, as described previously by Ho et al. [11]. Following the amplification of 16S rRNA, positive samples were selected for the detection of the virulence genes *cagA* and *vacA* using specific primers based on previous publications [12-15]. Polymerase chain reaction (PCR) was performed in a total volume of 12.5 µL containing 2× master mix with standard buffer (NEB Inc., Ipswich, MA) and 0.2 µM of forward and reverse primers. The primer sequences and annealing temperatures are described in Table 1. The PCR products were run on 1.2% agarose gel and visualized under an ultraviolet (UV) light using the GelDoc EZ Gel Imaging System (Bio-Rad, Hercules, CA).

**Table 1:** Primers for amplification in this study

Genes	Primers (5'-3')	Annealing temperature (°C)	Size (bp)	References
16S rRNA	Forward - CTGGAGAGACTAAGCCCTCC	55	110	[11]
	Reverse - ATTACTGACGCTGATTGTGC			
cagA (detection)	Forward - GATAACAGGCAAGCTTTTGAGG	52	349	[13]
	Reverse - CTGCAAAAGATTGTTTGGCAGA			
cagA (sequencing)	Forward - ACCCTAGTCGGTAATGGGTTA	56	Variable	[14]
	Reverse - GTAATTGTCTAGTTTCGC			
vacA (s1/s2)	Forward - ATGGAAATACAACAAACACAC	56	259/286	[15]
	Reverse - CTGCTTGAATGCGCCAAAC			
vacA (m1/m2)	Forward - CAATCTGTCCAATCAAGCGAG	57	570/642	[15]
	Reverse - GCGTCTAAATAATTCCAAGG			
vacA (i1)	Forward - GTTGGGATTGGGGGAATGCCG	53	426	[12]
	Reverse - TTAATTTAACGCTGTTTGAAG			
vacA (i2)	Forward - GTTGGGATTGGGGGAATGCCG	53	432	[12]
	Reverse - GATCAACGCTCTGATTTGA			

Cloning and sequencing analysis

The *cagA* genotypes and EPIYA motifs of *H. pylori* were determined with cloning and sequencing analysis. The 3′ variable region of *cagA* was amplified using One Taq® Hot Start 2× master mix with standard buffer (NEB Inc.). Subsequently, all fragments were cloned into the pGEM®-T easy vector system (Promega, Madison, WI). Recombinant clones were verified by *Eco*RI digestion followed by gel electrophoresis. Then, the recombinant plasmid was subjected to nucleotide sequencing using automated DNA sequencing at ATGC Co., Ltd. (Thailand). Nucleotide sequences were translated into amino acid sequences using the BioEdit program. Finally, the amino acid sequences were analyzed to determine the *cagA* genotypes and the EPIYA motif patterns.

Statistical analysis

Data were analyzed using the chi-square and ANOVA tests. A P-value of <0.05 was considered statistically significant.

## Results

Categories of diagnostic diseases

The diagnostic results of 250 patients who underwent endoscopy revealed that 110 (44%) had normal gastric mucosa, while 140 (56%) had gastrointestinal diseases. Among the 140 patients with gastrointestinal diseases, ages ranged from 25 to 87 years, with an average age of 61.83 ± 8.52 years. These 140 patients were categorized into specific gastrointestinal disease groups: 104 with gastritis, 21 with PUD, five with GC, and 10 with other gastrointestinal disorders, such as gastroesophageal reflux disease, Barrett's esophagus, polyps, and hiatal hernia. The results indicated that females were more frequently diagnosed with gastrointestinal diseases compared to males, with gastritis being the most common diagnosis in both genders. We divided the patients into three age groups: 21-40 years, 41-60 years, and over 60 years. A higher incidence of gastrointestinal diseases was observed in patients over the age of 60 compared to those under 60, with the majority in this age group being diagnosed with gastritis (Table 2).

**Table 2:** Endoscopic diagnosis in gastrointestinal patients according to sex and age group (N = 140)

Parameters	Clinical outcome (number (%))				Total
	Gastritis	Peptic ulcer	Gastric cancer	Other	
	n = 104	n = 21	n = 5	n = 10	N = 140
Sex					
Male	36 (34.6)	10 (47.6)	2 (40)	3 (30)	51 (36.4)
Female	68 (65.4)	11 (52.4)	3 (60)	7 (70)	89 (63.6)
Age (years)					
21-40	4 (3.9)	1 (4.8)	2 (40)	0 (0)	7 (5)
41-60	30 (28.8)	8 (38.1)	0 (0)	6 (60)	44 (31.4)
>60	70 (67.3)	12 (57.1)	3 (60)	4 (40)	89 (63.6)

Prevalence of *H. pylori*-positive infections in the patients

A total of 140 patients with gastrointestinal diseases were screened for *H. pylori* infection. Using 16S rRNA gene amplification, 45 (32.1%) patients tested positive for *H. pylori*. Among these 45 patients, 18 (40%) were male with a mean age of 60.64 ± 8.12 years, and 27 (60%) were female with a mean age of 60.68 ± 8.06 years. Endoscopic findings indicated that* H. pylori*-positive patients had gastritis and peptic ulcer diseases. However, *H. pylori* infection was not detected in samples from patients with gastric cancer and other gastrointestinal diseases. For *H. pylori*-positive patients, we assessed the relationship between sex and age with the different clinical diseases (Table 3). There were no statistically significant differences (P > 0.05).

**Table 3:** Distribution of H. pylori-positive patients with clinical outcomes according to sex and age (N = 45) P-values were calculated using the chi-square test. There were no statistical differences between sex and age with clinical outcomes (P > 0.05).

Parameters	Clinical outcome (number (%))		Total	P-value
	Gastritis	Peptic ulcer		
	n = 37	n = 8	N = 45	
Sex				0.524
Male	14 (37.8)	4 (50)	18 (40)	
Female	23 (62.2)	4 (50)	27 (60)	
Age (years)				0.08
20-40	2 (5.4)	0 (0)	2 (4.4)	
40-60	12 (32.4)	6 (75)	18 (40)	
>60	23 (62.2)	2 (25)	25 (55.6)	

Prevalence and distribution of *cagA* genotypes and EPIYA motifs

In this study, we investigated 45* H. pylori*-positive samples to determine the presence of the *cagA* gene. Forty (88.9%) of these samples tested positive for the *cagA* gene, while five (11.1%) were negative. Subsequently, we sequenced the 3′ variable region of the *cagA* gene. The most prevalent* cagA* genotype was the Western type (62.5%). Among Western-type samples, the majority exhibited the EPIYA-ABC motif; we also observed the EPIYA-ABCC, EPIYA-ACC, and EPIYA-CC motifs. Some *H. pylori* strains had East Asian type (37.5%), all of which had the EPIYA-ABD motif. The association between *cagA* genotypes and clinical outcomes was statistically significant, with the Western type being more prevalent in gastritis patients (Table 4).

**Table 4:** Distribution of cagA genotypes and EPIYA motif patterns in H. pylori-positive patients according to clinical outcomes *Data were analyzed using the chi-square test. P < 0.05 represents significant differences between cagA genotypes and clinical outcomes.

cagA status	Clinical outcome (number (%))		Total	P-value
	Gastritis	Peptic ulcer		
	n = 37	n = 8	N = 45	
cagA-	5 (13.5)	0 (0)	5 (11.1)	
cagA+	32 (86.5)	8 (100)	40 (89.9)	
cagA genotypes				0.014*
Western type	23 (71.9)	2 (25)	25 (62.5)	
ABC	19 (82.6)	2 (100)	21 (84)	
ABCC	1 (4.3)	0 (0)	1 (4)	
ACC	2 (8.6)	0 (0)	2 (8)	
CC	1 (4.3)	0 (0)	1 (4)	
East Asian type	9 (28.1)	6 (75)	15 (37.5)	
ABD	9 (28.1)	6 (75)	15 (37.5)	

Prevalence and distribution of *vacA* genotypes in *H. pylori *strains

We examined the presence of the* vacA* gene in all *H. pylori*-positive samples. All 45 samples had the *vacA* s and *vacA* m region, with the vacA s1 being the most frequent (43/45, 95.6%). The *vacA* m1 and *vacA* m2 alleles had similar frequencies, with 22 (48.9%) and 23 (51.1%) samples, respectively. The *vacA* i region was present in 44 of the 45 *H. pylori*-positive samples, with a high frequency of* vacA* i1 alleles (91.1%). We also examined the different combinations of *vacA* s, m, and i alleles in patients. The dominant *vacA* genotype combinations were s1m1i1 (46.7%) and s1m2i1 (44.5%). There were no statistical differences between the *vacA* genotypes and clinical outcomes (P > 0.05) (Table 5).

**Table 5:** Distribution of vacA genotypes in H. pylori-positive patients according to clinical outcomes P-values were calculated using the chi-square test. There were no statistical differences between vacA genotypes with clinical outcomes (P > 0.05).

Genotypes	Clinical outcome (number (%))		Total
	Gastritis	Peptic ulcer	
	n = 37	n = 8	N = 45
vacA s			
s1	35 (94.6)	8 (100)	43 (95.6)
s2	2 (5.4)	0 (0)	2 (4.4)
vacA m			
m1	20 (54.1)	2 (25)	22 (48.9)
m2	17 (45.9)	6 (75)	23 (51.1)
vacA i			
i1	33 (89.2)	8 (100)	41 (91.1)
i2	3 (8.1)	0 (0)	3 (6.7)
Combine			
s1m1	1 (2.7)	0 (0)	1 (2.2)
s1m1i1	19 (51.4)	2 (25)	21 (46.7)
s1m2i1	14 (37.8)	6 (75)	20 (44.5)
s1m2i2	1 (2.7)	0 (0)	1 (2.2)
s2m2i2	2 (5.4)	0 (0)	2 (4.4)

## Discussion

The prevalence of *H. pylori* infection and the genetic variation of its virulence genes contribute to the varying risk of GC in different regions. Numerous studies have investigated the diversity of these genes due to their association with an increased risk of GC [16]. Previous studies have demonstrated that* H. pylori* strains carrying *cagA* are more virulent than those that do not carry *cagA*, leading to higher levels of gastric mucosal inflammation, severe atrophic gastritis, and the development of GC [2]. We observed a high prevalence of* H. pylori* strains carrying *cagA*, approximately 90%, consistent with findings from Northeast Thailand and South Korea, with approximately 98% and 96% *cagA*-positive strains, respectively [17,18]. Recent studies have emphasized the significance of the diversity of CagA sequences, particularly in the tyrosine phosphorylation sites within the unique five amino acid repeats known as EPIYA motifs. These motifs play crucial roles in regulating cell spreading, migration, and adhesion, primarily through their interaction with protein-tyrosine phosphatase (SHP2).

Studies have classified *cagA* sequences into two main types: Western and East Asian. East Asian type has been reported to be more virulent than the Western type due to its stronger binding affinity for SHP2 and greater morphological transforming activities, leading to higher mucosal inflammation and increased risk of gastric carcinogenesis [5]. We observed a high prevalence of Western-type *cagA*, consistent with previous studies in South Asian and Southeast Asian populations [19,20]. Most of the patients with gastritis in our study had Western-type* H. pylori* strains carrying the EPIYA-ABC motif, with some also exhibiting multiple EPIYA-C segments. Previous studies have indicated that patients infected with *H. pylori* strains carrying multiple EPIYA-C segments are more likely to develop gastric diseases and have a higher risk of GC compared with those infected with strains containing a single EPIYA-C segment. Specifically, patients diagnosed with gastric ulcers and cancer have been reported to harbor *H. pylori* strains carrying *cagA* with the EPIYA-ABCCC and EPIYA-ABCC motifs, respectively. The increase in the number of phosphorylation sites in the C-terminus of CagA may be associated with the carcinogenic potential of *H. pylori*, leading to greater inflammatory cell infiltration [21,22]. However, some studies have suggested that a single EPIYA-C motif phosphorylation is sufficient for cellular perturbation and a pro-inflammatory response to *H. pylori* infection, independent of the number of *cagA* EPIYA-C motifs [23].

We found the presence of the* vacA* gene in all *H. pylori*-infected patients and noted a variety of genotypes. Specifically, we found a high prevalence of the *vacA* s1 and i1 alleles among patients with* H. pylori* infection, with the *vacA* m1 and m2 alleles being nearly equally represented. This finding is consistent with patterns observed in Asian countries, where most *H. pylori *strains contain the *vacA* s1 and i1 alleles. Numerous studies conducted in Western countries have demonstrated a correlation between the *H. pylori* *vacA *genotypes and clinical outcomes. Specifically, it has been indicated that individuals infected with *H. pylori* carrying *vacA* s1m1 face an elevated risk of PUD and/or GC compared to those infected with *H. pylori* carrying* vacA* s2m2 [24]. Moreover, patients infected with* H. pylori* carrying the *vacA* i1 allele are at an increased risk for the progression of gastric precancerous lesions and gastric carcinoma [25,26]. In East Asian countries, elucidating the relationship between *H. pylori* *vacA* s-region genotypes and gastric carcinoma has been challenging, as nearly all strains are *vacA* s1. In contrast, variability is more apparent in the *vacA* m region: m1 alleles predominate in Japan and South Korea, where GC rates are high, while m2 alleles are more prevalent in Southeast Asian countries, such as Vietnam [27-29]. This variability suggests that the m region may play a role in regional differences in disease patterns.

In addition to geographic differences, ethnic differences contribute to the variation of genotypes. In previous studies, *H. pylori *strains containing* the vacA* m2 allele were predominantly found in Southeast Asian countries, where the incidence of gastric cancer is low. Our study showed a similar prevalence of both m1 and m2 strains among *H. pylori*-infected patients. This likely reflects the diverse demographic composition of the study participants, including individuals of mixed ethnic backgrounds, such as those of Thai-Chinese descent. As a result, we observed a mixture of *vacA* m genotypes in the *H. pylori* strains isolated from patients. Despite reports suggesting that the *vacA* m1 strain is more virulent than m2, no consistent association between the *vacA* m genotype and clinical outcomes has been observed in Asian countries. Furthermore, we demonstrated that the s1m1 strain with the i1 allele was dominant. Studies conducted in East Asia have revealed a significant association between the *vacA* i1 allele and the development of PUD and GC, with i1 considered a biomarker for *H. pylori*-related gastrointestinal diseases [3,12,30]. However, several studies have noted a high frequency of the* vacA* i1 allele in *vacA* s1m1 strains, yet clinical outcomes have shown variability among these studies [3]. Therefore, the role of the *vacA* i genotype as a definitive marker for GC development remains inconclusive.

Finally, we had limitations due to the small sample size in this study. At our hospital, we observed a low incidence of GC, consistent with the relatively low risk of developing GC in Thailand compared to other Asian countries. In our study, we did not detect *H. pylori* infections among GC patients. Diagnostic results also revealed varying sample size between patients with PUD and those with gastritis, as well as differences in the prevalence of* H. pylori* infection. Consequently, we were unable to obtain a similar sample size in each patient group, potentially leading to unclear correlations between *cagA* and *vacA* genotypes and clinical disease outcomes. Therefore, further studies with larger numbers of patients will be necessary to clarify our findings.

## Conclusions

The present study investigated the *cagA* and *vacA* genotypes of *H. pylori *isolated from gastrointestinal patients in Central Thailand. We found a high prevalence of the Western-type *cagA* with the EPIYA-ABC motif. The dominant *vacA* genotypes observed were s1 and i1, with m1 and m2 also being prominent. Although we did not establish a clear association between *cagA* EPIYA motifs, *vacA* genotypes, and clinical outcomes, these findings could be crucial for clinical and epidemiological surveys aimed at better understanding the disease pathology.
